# Luteolin Ameliorates Methamphetamine-Induced Podocyte Pathology by Inhibiting Tau Phosphorylation in Mice

**DOI:** 10.1155/2022/5909926

**Published:** 2022-03-24

**Authors:** Jiuyang Ding, Yuanhe Wang, Zhuo Wang, Shanshan Hu, Zhu Li, Cuiyun Le, Jian Huang, Xiang Xu, Jiang Huang, Pingming Qiu

**Affiliations:** ^1^Department of Forensic Medicine, Guizhou Medical University, Guiyang 550004, Guizhou, China; ^2^School of Forensic Medicine, Southern Medical University, Guangzhou 510515, Guangdong, China; ^3^Department of Infertility and Sexual Medicine, The Third Affiliated hospital of Sun Yat-sen University, Guangzhou 510630, Guangdong, China; ^4^Good Clinical Practice Center, Affiliated Hospital of Zunyi Medical University, Zunyi 563003, China; ^5^School of Forensic Medicine, Wannan Medical College, Wuhu 241000, Anhui, China

## Abstract

Methamphetamine (METH) can cause kidney dysfunction. Luteolin is a flavonoid compound that can alleviate kidney dysfunction. We aimed to observe the renal-protective effect of luteolin on METH-induced nephropathies and to clarify the potential mechanism of action. The mice were treated with METH (1.0–20.0 mg/kg/d bodyweight) for 14 consecutive days. Morphological studies, renal function, and podocyte specific proteins were analyzed in the chronic METH model in vivo. Cultured podocytes were used to support the protective effects of luteolin on METH-induced podocyte injury. We observed increased levels of p-Tau and p-GSK3*β* and elevated glomerular pathology, renal dysfunction, renal fibrosis, foot process effacement, macrophage infiltration, and podocyte specific protein loss. Inhibition of GSK3*β* activation protected METH-induced kidney injury. Furthermore, luteolin could obliterate glomerular pathologies, inhibit podocyte protein loss, and stop p-Tau level increase. Luteolin could also abolish the METH-induced podocyte injury by inactivating GSK3*β*-p-Tau in cultured podocytes. These results indicate that luteolin might ameliorate methamphetamine-induced podocyte pathology through GSK3*β*-p-Tau axis.

## 1. Introduction

Methamphetamine (METH), an amphetamine type stimulator (ATS), can stimulate extensive brain areas leading to neurological dysfunction and neurodegeneration [1, 2]. METH is a strong stimulator of several neurotransmitters, especially dopamine and serotonin which might lead to hyperexcitability, hallucinogenic effect, and hypersexuality. Besides the central nervous system effect, METH might induce dysfunctions among other organs including the liver, heart, and kidney [3–5].

Glomerular disease is the main cause of chronic kidney disease. In 2017, the chronic kidney disease case was about 7 million [6]. Thus, podocyte and glomerular research of therapeutic strategies for chronic progressive kidney diseases is the hotspot worldwide. The nephrotoxicity of METH probably attributes to hyperthermia, oxidative stress, inflammatory cell infiltration, vasopressin secretion, and impaired energy metabolism [7–9]. Acute METH intoxication could induce tricarboxylic acid cycle dysfunction and fatty acid metabolism, and the metabolic intermediates could be potential biomarkers of METH intoxication [8]. However, the METH-induced nephropathy has not been studied previously.

Luteolin (Lut), a flavonoid compound, attracts much attention in treating vascular inflammation, dopaminergic neurons degeneration, and tumors [10–12]. However, protective effects of luteolin on METH-induced nephropathy remain unclear.

The microtubule-associated protein Tau, a member of the microtubule binding protein family, is expressed in multiple organs or tissues including the brain, skeletal muscle, heart, kidney, testis, and pancreas [13–15]. A large amount of research has been focused on the functions of Tau in the central nervous system especially in neurons [16], where Tau functions as a microtubule stabilizer [17]. Accumulation of Tau, particularly the phosphorylated form of Tau (p-Tau), in neurons is the hallmark of Alzheimer's disease [18]. Also, Tau deficiency could lead to Parkinson's disease through iron transport dysfunction [19]. Recently, Tau in podocytes has attracted much attention of nephrologists. The roles of Tau in podocyte were mainly associated with podocyte morphology and physiological functions. Similar to that in neurons, p-Tau plays a key role in podocyte microtubule stability. Studies have shown that p-Tau leads to podocyte cell body shrinkage and foot process effacement in an adriamycin-induced mice model with chronic kidney disease [20]. However, the role of Tau phosphorylation in METH-induced podocyte injury remains unclear. We hypothesize that METH-induced Tau phosphorylation might mediate podocyte morphological abnormality, podocyte specific protein loss, and dysfunction.

In our study, we demonstrated that METH could lead to glomerular fibrosis, inflammation, and podocyte specific protein loss. All the podocyte lesions were accompanied by increasing p-Tau. Inhibiting Tau phosphorylation by blocking Tau kinase GSK3*β* could alleviate METH-induced nephropathies. Similarly, luteolin could block METH-induced kidney injury through p-Tau inhibition. METH could lead cultured podocytes overactive to GSK3*β*, and hyperphosphorylation of Tau resulted in podocyte damage. Luteolin treatment can override GSK3*β* activation, Tau phosphorylation, and exert a cytoprotective effect against METH in cultured podocytes. These results provide a promising drug candidate for avoiding chronic METH-induced kidney injury.

## 2. Materials and Methods

### 2.1. Animals

C57BL/6 J mice (6–8 weeks old, male, 20–24 g) were provided by Laboratory Animal Center of Southern Medical University (Guangzhou, China). All animals were housed in a standard environment with standard food and water. All procedures on the animals were carried out according to the guide from National Institutes of Health and were approved by the Southern Medical University Animal Care and Use Committee.

### 2.2. Drug Treatment and Experimental Groups

METH (purity >99%, National Institutes for Food and Drug Control, Guangzhou, China) was administered intraperitoneally, as given in Table 1, to establish the chronic METH mice model [21]. The administration started with low dosage and ended with high challenge doses simulating the progression of METH doses observed in human usage. Luteolin (Push BioTechnology, Chengdu, China, purity >96%) in dimethyl sulfoxide (DMSO) was administered by gavage. The dose of luteolin was set at 100 mg/kg bodyweight/day according to the previous studies [22, 23]. Lithium chloride (Solarbio Life science, Beijing, China) was used as an inhibitor of GSK3*β*.

After 7 days acclimation, 36 mice were randomly divided into six groups (6 per group).

#### 2.2.1. Con

Saline was administered intraperitoneally in place of METH. DMSO was administered (from 1^st^ day to 21^st^ day) via gavage in place of luteolin;

#### 2.2.2. METH + Lut

Luteolin (100 mg/kg bodyweight/day, once daily) was administered via gavage for 3 weeks (from 1^st^ day to 21^st^ day), and METH (dissolved in saline) was assessed for the last 2 weeks (from 8^th^ day to 21^st^ day, as given in Table 1)

#### 2.2.3. METH

METH was administered intraperitoneally for two weeks (from 8^th^ day to 21^st^day, as given in Table 1). DMSO was administered (from 1^st^ day to 21^st^ day) via gavage in place of luteolin.

#### 2.2.4. Lut

Luteolin (100 mg/kg bodyweight/day, once daily) was administered via gavage for 3 weeks (from 1^st^ day to 21^st^ day), and saline was administered intraperitoneally in place of METH.

#### 2.2.5. LiCl + METH

Lithium chloride (40 mg/kg bodyweight/day, once daily) was administered intraperitoneally for 3 days (on the 1^st^ day, 7^th^ day, and 14^th^ day), and METH was injected for the last 2 weeks (from 8^th^ day to 21^st^day, as given in Table 1).

#### 2.2.6. LiCl

Lithium chloride (40 mg/kg bodyweight/day, once daily) was administered intraperitoneally for 3 days (on the 1^st^ day, 7^th^ day, and 14^th^ day), and saline was administered intraperitoneally in place of METH. The second day after METH Injection, the mice were anesthetized with ketamine (120 mg/kg bodyweight, Gutian Pharmaceutical Co., Ltd., Fujian, China) and xylazine (8 mg/kg bodywight, Topscience Co., Ltd., Shanghai, China). Blood samples were acquired for serum creatinine (SCr) and blood urea nitrogen (BUN) analyses using Elisa kits (Cat#EPBIO 1590 and Cat#HPBIO 1589, HEPENG Biological, Shanghai, China), respectively. The 24 h urine was collected for albumin quantification using the albumin ELISA kit (Cat#CSB-E13878, CUSABIO Tech, China). Then, cold saline was perfused from the left ventricle for 5 min before mice kidney tissues were collected.

### 2.3. Hematoxylin-Eosin (H&E) and Masson's Trichrome Staining

Kidneys were fixed in 4% paraformaldehyde (Beyotime Biotechnology, Shanghai, China) and embedded in paraffin blocks. Three (3) *μ*m sections were stained with H&E. Masson's trichrome stain was performed with iron hematoxylin, ponceau, and toluidine blue in order to stain the nucleus, muscular fibers, and collagen fibers, respectively. All stained sections were observed using a fluorescence microscope equipped with a camera (Zeiss Metafer Z2, Germany).

### 2.4. Immunohistochemistry (IHC) Staining

Kidney sections (3 *μ*m) were dewaxed, washed in PBS before use. After inhibiting endogenous peroxidases and blocking, sections were incubated with various primary antibodies including phosphorylated GSK3*β* (p-GSK3*β*), p-Tau, synaptopodin, and podocalyxin-like protein 1 (Table 2) overnight at 4°C. Then, the signals were visualized using the diaminobenzidine (DAB) kit (Cat#CW2069, CW Bio, China). Images were captured using a fluorescence microscope (Zeiss Metafer Z2, Germany). Signal intensity was assessed using Image J version 1.40 (National Institutes of Health, Bethesda, MD, USA). The number of staining-positive cells was counted in a blind manner. Three kidney sections in each animal, and five high magnification microscopic fields per section were analyzed.

### 2.5. TUNEL Staining

The 3 *μ*m kidney sections were dewaxed and washed in PBS before use. Then, the sections were processed according to the protocols of the TUNEL assay kit (Cat#ab206386, Abcam, Cambridge, USA). Briefly, the sections were reacted with the terminal deoxynucleotidyl transferase (TDT) labeling reaction mixture for 1.5 hours and then covered with DAB solution for 15 minutes. The nuclei were stained with hematoxylin. Images were captured using a fluorescence microscope (Zeiss Metafer Z2, Germany). The TUNEL positive cell number count was performed in a blind manner.

### 2.6. Transmission Electron Microscope (TEM) and Scanning Electron Microscope (SEM) Analyses

The TEM samples of kidney tissues were performed as described in our previous studies [24]. Briefly, tissues were fixed in 2.5% glutaraldehyde (Millipore Sigma, Burlington, MA, USA) at 4°C for 8 hours, embedded in Epon resin (Polyscience, Inc., Eppelheim, Germany), and then sectioned using a ultramicrotome (EM UC7, Leica, Wetzlar, Germany). Images were captured using an electron microscope (Tecnai G2, FEI, CA, USA) at 120 kV of voltage.

For SEM analysis, the kidney samples were fixed using osmium tetroxide and then coated with gold. Samples were observed using a FEI Quanta 250 FEG scanning electron microscope. For both TEM and SEM analyses, ten glomeruli per animal were observed.

### 2.7. In Vitro Experiment

Briefly, mouse podocytes were isolated from mice weighing 20–22 g according to the method described earlier [25]. Mice were anesthetized using 120 mg/kg ketamine (Gutian Pharmaceutical Co., Ltd., Fujian, China) and 8 mg/kg xylazine (Topscience Co., Ltd., Shanghai, China) and then perfused with saline followed by Dynabead (Thermo Fisher Scientific, Waltham, MA, USA) through the left ventricle for 2 min. Then, the kidneys were acquired and cut into pieces followed by digestion with collagenase IV (Chemical book, Beijing, China) at 37°C for 15 min. The mouse podocytes were cultured in DMEM (Thermo Fisher Scientific, Waltham, MA, USA) containing IFN-*γ* (10 *μ*/mL, Topscience Co., Ltd., Shanghai, China) at 33°C and then polarized at 37 C. The mouse podocytes were cultured with or without METH (0.2 mM), luteolin (50 *μ*M), and LiCl (10 mM).

### 2.8. Western Blot Analysis

Mouse podocytes were lysed with RIPA buffer (Cell Signaling Technology, Danvers, MA, USA) containing protease inhibitor (Topscience Co., Ltd., Shanghai, China). Briefly, the targeted proteins were quantified by using antibodies against phosphorylated GSK3*β* (p-GSK3*β*), GSK3*β*, p-Tau, synaptopodin, and podocalyxin-like protein 1. Actin was used as the internal indicator [26–28].

### 2.9. Statistical Analysis

All data are expressed as the mean (*M*) ± standard deviation (SD). All statistical analyses were performed using one-way ANOVA followed by Bonferroni post hoc analyses using SPSS version 20.0 (IBM corporation, Armonk, NY, USA). The threshold of *P* < 0.05 was assumed statistically significant.

## 3. Results

### 3.1. Rescue Treatment with Luteolin Alleviates Glomerular Pathology and Inflammation in Experimental Chronic METH Nephropathy

**Figure 1:**
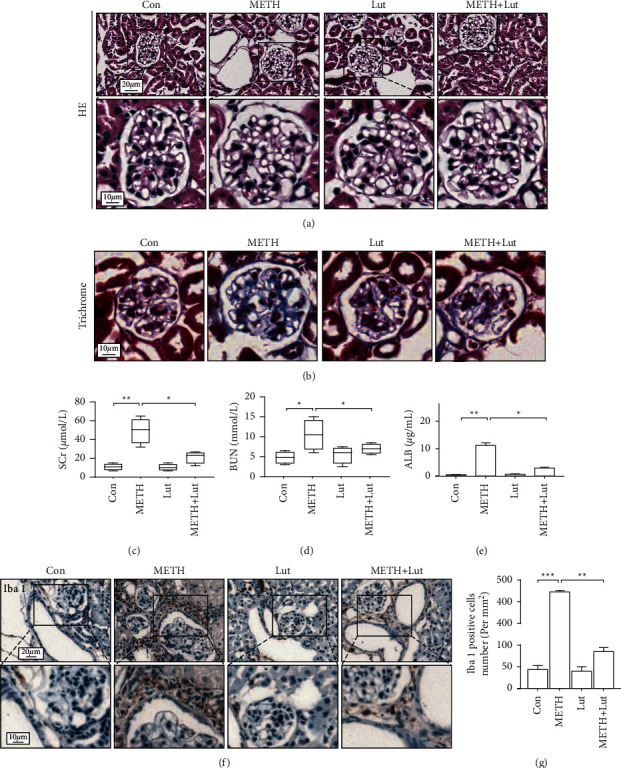
Glomerular pathology after chronic methamphetamine exposure in each group. (a) Representative images of H&E staining of mouse kidneys. (b) Representative images of Masson's trichrome staining of mouse kidneys. (c) SCr level in each group mice. (d) BUN level in each group mice. (e) Urine albumin level in each group mice. (f) Immunohistochemistry staining of Iba-1 in mouse kidneys. (g) Analysis of number of Iba-1 positive cells in each group; ^*∗*^*p* < 0.05, ^*∗∗*^*p* < 0.01, and ^*∗∗∗*^*p* < 0.001 by one-way ANOVA and Bonferroni post hoc analyses. *n* = 6 per group.

We observed normal glomerular morphology with H&E staining in the saline control group and luteolin only group. After 14 days of METH exposure, mouse glomeruli exhibited morphological abnormality including expansion of Bowman's capsule, glomerular cell nuclear fragmentation, and mesangial proliferation. In contrast, the glomeruli in the Lut + METH group ameliorated these glomerular changes compared with the METH group (Figure 1(a)). Furthermore, METH-induced fibrosis in kidney, especially in the glomeruli. Pretreatment with Lut alleviated renal fibrosis (Figure 1(b)). Both SCr and BUN levels in the METH group were higher than that in control groups, and Lut treatment could prevent the increase in SCr levels and even normalize the BUN level after METH exposure (Figures 1(c) and 1(d)). Lut also alleviated albuminuria induced by METH (Figure 1(e)). The Iba-1 (a macrophage marker) staining showed several macrophages localized around the vascular tissues and glomeruli in the control group. Numerous macrophages infiltrated into the renal interstitium and glomeruli after METH treatment. Luteolin could reverse the macrophage number increase induced by METH (Figures 1(f) and 1(g)).

### 3.2. Luteolin Inhibited Tau Phosphorylation Induced by METH

**Figure 2:**
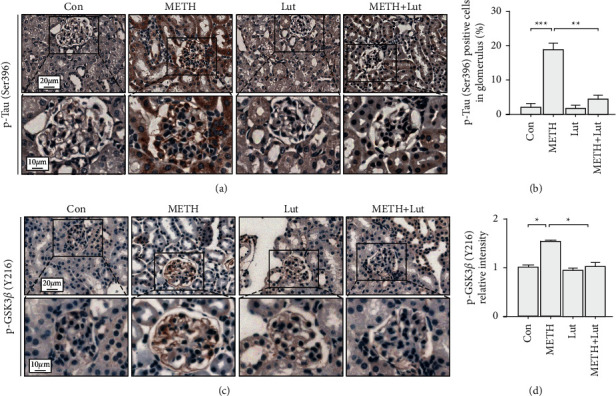
Luteolin reversed Tau phosphorylation induced by methamphetamine. (a) Representative micrographs of p-Tau (Ser396) immunohistochemistry staining in mouse kidneys. (b) Analysis of p-Tau (Ser396) positive cells number in each group. (c) Representative micrographs of p-GSK3*β* (Y216) immunohistochemistry staining in mouse kidneys. (d) Analysis of p-GSK3*β* (Y216) intensity in each group; ^*∗*^*p* < 0.05, ^*∗∗*^*p* < 0.01, and ^*∗∗∗*^*p* < 0.001 by one-way ANOVA and Bonferroni post hoc analyses. *n* = 6 per group.

To explore the METH on Tau phosphorylation, we stained p-Tau (Ser396) in kidney tissues. In the control group, few p-Tau positive cells were found. An intense p-Tau staining was observed in the METH group. Luteolin pretreatment reduced p-Tau positive cells induced by METH (Figures 2(a) and 2(b)). Then, we test the Tau kinase GSK3*β* level in each group. The result showed that there was barely any p-GSK3*β* (Y216) staining in the control group, whereas METH treatment increased the p-GSK3*β* intensity. Luteolin treatment could reverse the p-GSK3*β* intensity increase induced by METH (Figures 2(c) and 2(d)).

### 3.3. Luteolin Rescued Podocyte-Specific Protein Loss Induced by METH

**Figure 3:**
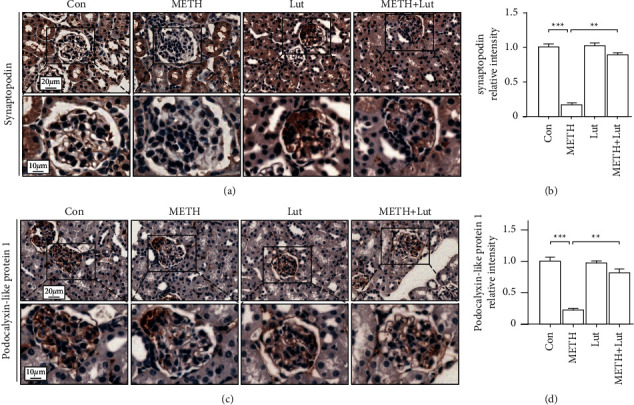
Luteolin rescued methamphetamine-induced podocyte protein loss. (a) Representative micrographs of synaptopodin immunohistochemistry staining in mouse kidneys. (b) Analysis of synaptopodin intensity in each group. (c) Representative micrographs of podocalyxin-like protein 1 immunohistochemistry staining in mouse kidneys. (d) Analysis of podocalyxin-like protein 1 intensity in each group; ^*∗*^*p* < 0.05, ^*∗∗*^*p* < 0.01, and ^*∗∗∗*^*p* < 0.001 by one-way ANOVA and Bonferroni post hoc analysis. *n* = 6 per group.

Next, we focused on the podocyte pathology induced by METH. We found the podocyte protein synaptopodin and podocalyxin-like protein 1 decreased after METH treatment. Pretreatment of Lut reversed the podocyte protein loss induced by METH (Figure 3).

### 3.4. Luteolin Inhibited Glomerular Ultrastructural Pathology Induced by METH

**Figure 4:**
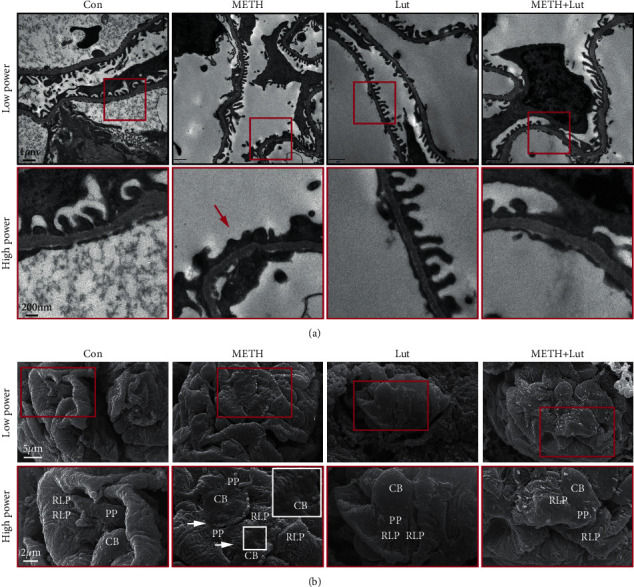
Luteolin rescued methamphetamine-induced glomeruli ultrastructure deterioration. (a) TEM analysis of podocyte foot process morphology after methamphetamine treatment. Red arrows indicate podocyte foot process fusion. (b) SEM analysis of podocyte surface structure. CB, cell body; PP, primary process; RLP, ridge-like prominence.

To observe the ultrastructure of podocytes, we conducted TEM and SEM analyses. TEM results showed the normal podocyte foot process in saline control and Lut only groups. Rescue treatment with Lut attenuated the podocyte foot process fusion induced by METH (Figure 4(a)). To verify the TEM results, we observed the architecture of podocyte using SEM. Results showed the podocyte cell body (CB), primary foot process (PP), and ridge-like prominence (RLP) were normal in the Con and Lut group. However, we found that the bleb-like protrusions were on the podocyte cell body, the CB and PP were flattened, and the PP was morphologically abnormal or disappeared after METH treatment. In the Lut + METH group, the CB, PP, and RLP pathology were attenuated, except there were microvilli on the CB or PP (Figure 4(b)).

### 3.5. LiCl Restored Podocyte Protein Loss in METH-Induced Podocyte Injury

**Figure 5:**
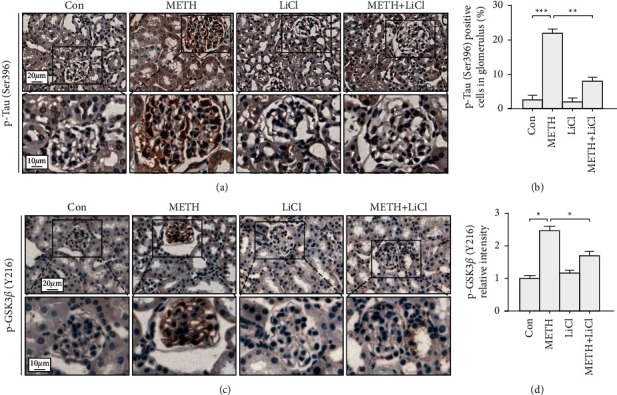
Lithium chloride inhibited the GSK3*β*-Tau axis. (a) Representative micrographs of p-Tau (Ser396) immunohistochemistry staining in mouse kidneys. (b) Analysis of p-Tau (Ser396) positive cells in each group. (c) Representative micrographs of p-GSK3*β* (Y216) immunohistochemistry staining in mouse kidneys. (d) Analysis of p-GSK3*β* (Y216) intensity in each group; ^*∗*^*p* < 0.05, ^*∗∗*^*p* < 0.01, and ^*∗∗∗*^*p* < 0.001 by one-way ANOVA and Bonferroni post hoc analysis. *n* = 6 per group.

To observe the effect of inhibiting GSK3*β* on METH-induced nephropathy, we tested the podocyte specific protein level after LiCl (GSK3*β* inhibitor) treatment. We found LiCl could reverse p-GSK3*β* Y216 (the activated form of GSK3*β*)/GSK3*β* increase induced by METH (Figures 5(a) and 5(b)). Next, we tested the p-Tau (a substrate of GSK3*β*) level after LiCl treatment. Results showed LiCl reduced the p-Tau positive cell number induced by METH (Figures 5(c) and 5(d)).

### 3.6. LiCl Attenuated Podocyte Injury in the METH Mice Model

**Figure 6:**
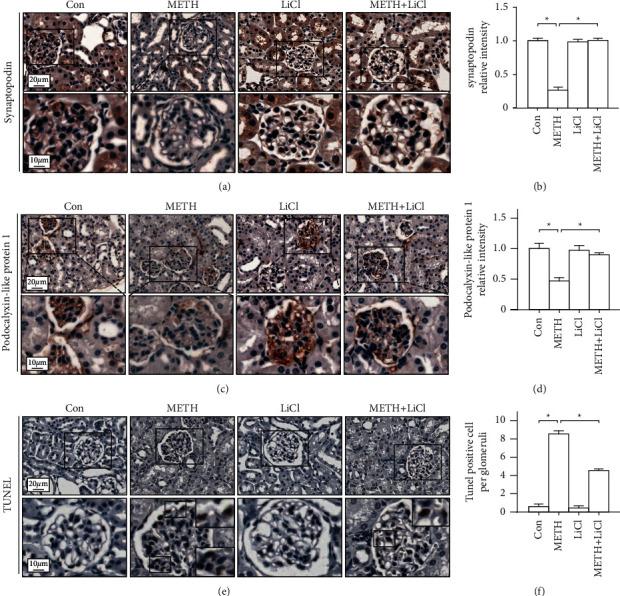
Lithium chloride rescued methamphetamine-induced podocyte protein loss and podocyte apoptosis. (a) Representative micrographs of synaptopodin immunohistochemistry staining in mouse kidneys. (b) Analysis of synaptopodin intensity in each group. (c) Representative micrographs of podocalyxin-like protein 1 immunohistochemistry staining in mouse kidneys. (d) Analysis of podocalyxin-like protein 1 intensity in each group. (e) Representative micrographs of TUNEL staining in mouse kidneys. (f) Analysis of TUNEL positive cells in each group; ^*∗*^*p* < 0.05, ^*∗∗*^*p* < 0.01, and ^*∗∗∗*^*p* < 0.001 by one-way ANOVA and Bonferroni post hoc analysis. *n* = 6 per group.

We conducted an immunohistochemistry (IHC) study on synaptopodin and podocalyxin-like protein 1, both of which are podocyte-specific proteins, to look into the effect of LiCl on podocyte. Synaptopodin and podocalyxin-like protein 1 were decreased after METH intoxication, and LiCl could reverse the decrease (Figures 6(a)–6(d)). Next, we tested the apoptotic cells in glomerulus using TUNEL staining. Result showed that LiCl could rescue the cell apoptosis induced by METH (Figures 6(e) and 6(f)).

**Figure 7:**
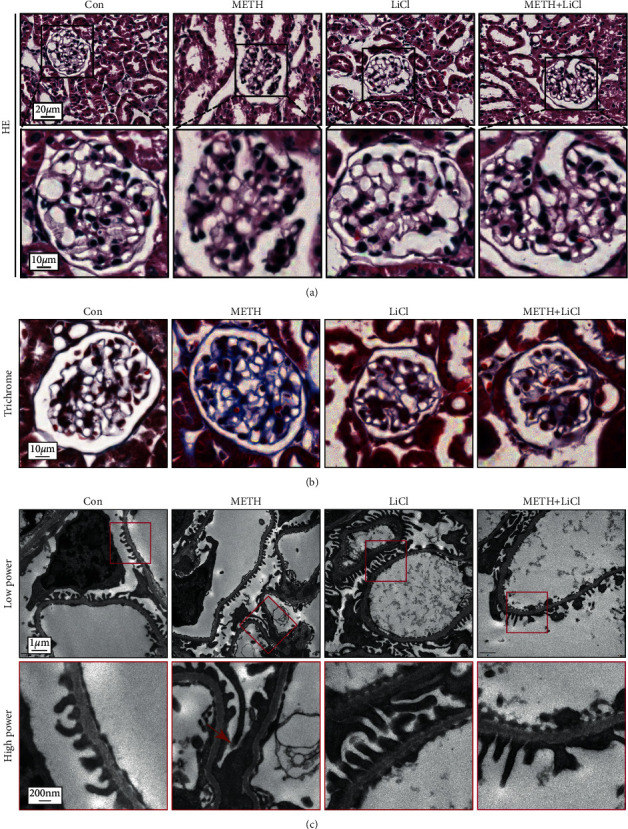
Lithium chloride attenuated podocyte injury in the methamphetamine mice model. (a) Representative images of H&E staining of mouse kidneys. (b) Representative images of Masson's trichrome staining of mouse kidneys. (c) TEM analysis of podocyte foot process morphology, red arrows indicate podocyte foot process fusion.

H&E and Masson's trichrome staining images showed LiCl could rescue the nephropathy and renal fibrosis induced by METH (Figures 7(a) and 7(b)). Furthermore, TEM images showed that chronic METH-induced podocyte foot process effacement, and LiCl evidently protected these ultrastructural pathologies (Figure 7(c)).

### 3.7. Luteolin Buffered In Vitro Podocyte Injury Induced by METH

**Figure 8:**
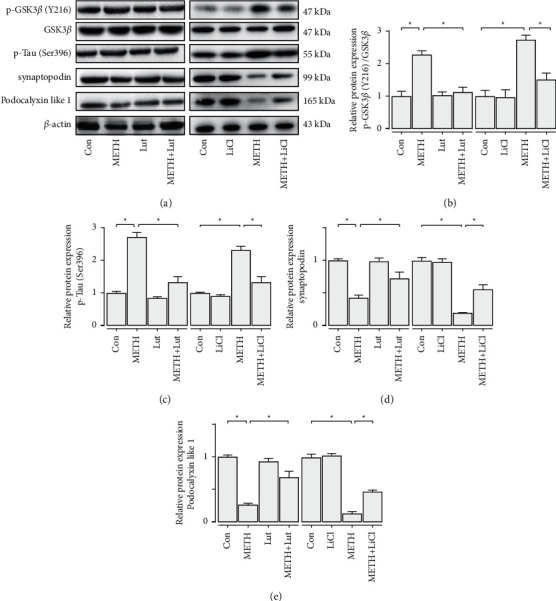
Luteolin buffered podocyte injury induced by METH in vitro. (a) Western blot analysis of p-GSK3*β* (Y216), GSK3*β*, p-Tau (Ser396), synaptopodin, and podocalyxin-like protein 1 in cultured podocytes. (b) Statistical analysis of relative level p-GSK3*β* (Y216)/GSK3*β* in each group. (c) Statistical analysis of relative level p-Tau (Ser396) in each group. (d) Statistical analysis of relative level synaptopodin in each group. (e) Statistical analysis of relative level of podocalyxin-like protein 1 in each group; ^*∗*^*p* < 0.05, ^*∗∗*^*p* < 0.01, and ^*∗∗∗*^*p* < 0.001 by one-way ANOVA and Bonferroni post hoc analysis. *n* = 3 per group.

To demonstrate the METH effect on podocyte directly, we conducted an in vitro experiment, in which METH directly led to Tau phosphorylation, GSK3*β* activation, and podocyte specific protein loss. Lut could reverse these changes. Moreover, LiCl treatment inhibited phosphorylation of GSK3*β* and Tau. Furthermore, LiCl abolished METH-induced podocyte specific protein loss in cultured podocytes (Figure 8).

## 4. Discussion

Here, we demonstrated chronic METH exposure may increase p-GSK3*β* and p-Tau levels and lead to glomerulopathy, renal dysfunction, renal fibrosis, and podocyte pathology. Inhibiting GSK3*β* activation using LiCl could reverse p-Tau upregulation and related nephropathies. A flavonoid compound luteolin rescued METH-induced nephropathy potentially through p-Tau dependency. Moreover, luteolin could protect cultured podocyte through the GSK3*β*-p-Tau pathway in an in vitro METH model. To our knowledge, this experiment is the first report on the renoprotective effect of luteolin in METH-induced podocyte pathology. Thus, this study could provide a basic animal research of luteolin application to clinical use in drug-induced nephropathy in the future.

METH users tend to have glomerulonephritis in later years according to a clinical survey [29]. In vivo experiments showed METH could lead to renal dysfunction, generally characterized as an increase in serum creatinine levels. Subacute METH treatment could trigger an increase of 8-hydroxydeoxyguanosine (8-OH-dG), an oxidative stress marker of DNA, in glomerular cells and renal tubular epithelial cells [30]. Consistent with these studies, our data revealed several apoptotic cells in glomeruli after METH intoxication (Figures 6(e) and 6(f)). Moreover, renal fibrosis and podocyte pathology were only observed partially due to our chronic METH model, which was just 14 days. We anticipate that the nephropathy and renal function would be more deteriorated in a long-term use of the chronic METH model than the subacute model.

In this study, the activated forms of GSK3*β* and p-Tau were upregulated mainly in the podocytes after METH exposure (Figure 2). Previous studies showed that p-GSK3*β* and p-Tau were concurrently increased accompanying renal dysfunction and nephropathy after adriamycin injury [20]. The Tau protein, as a microtubule stabilizer, plays an important role in podocyte morphology and function [31]. Tau is necessary for organelle function and protein transportation, both of which are critical to maintain cellular function [32, 33]. The phosphorylation of Tau disables its function and leads to microtubule destruction and destabilization [34]. Thus, we expected to see that METH-induced Tau phosphorylation might trigger dysfunction and pathological changes in podocyte. In line with this speculation, we observed podocyte specific protein loss and morphological abnormalities in the METH-treated group (Figures 3 and 4). Collectively, we would like to propose that the chronic METH-induced podocyte pathology was p-Tau dependent, and p-Tau level upregulation might be due to its kinase GSK3*β* activation.

METH has been shown to trigger oxidative stress and inflammation in multiple organs [9, 24]. Recent studies have shown that luteolin possess anti-inflammatory effects in neurodegenerative diseases, arthritis, and hepatopathy [22, 35]. Since large amount of METH-induced hormesis resembles aging or senescence-related degenerative disorder through redox and inflammation dependent, it is reasonable to observe that luteolin prevented macrophages infiltration around the glomerulus induced by METH [36, 37]. Moreover, we showed that luteolin prevented GSK3*β* activation and p-Tau level increase in the chronic METH model. In addition, luteolin prevented METH-induced renal fibrosis and podocyte pathology. Collectively, we speculate that the protective effect of luteolin on nephropathy may be through inhibiting the Tau phosphorylation and anti-inflammatory effect. Thus, it was not surprising to find that podocyte morphology and function were recovered when the p-Tau level was decreased.

Based on the results that luteolin could prevent METH-induced GSK3*β* activation, Tau phosphorylation, and nephropathy, we hypothesize that inhibiting GSK3*β* activation could also alleviate nephropathy. To verify this hypothesis, we used LiCl to inhibit GSK3*β* directly and demonstrated the protective benefit of LiCl on METH-induced nephropathy. Studies have shown that LiCl protects nephropathy by enhancing autophagy in the acute kidney injury mice model [38]. Accumulating data also indicate that LiCl could diminish p-Tau level through GSK3*β*, resulting in podocyte pathology amelioration in adriamycin-induced nephropathy [20]. In accordance with those findings, we found LiCl prevented p-Tau level increase and rescued METH-induced nephropathy.

We speculate that luteolin ameliorates METH-induced podocyte pathology by regulating Tau phosphorylation, which might trigger podocyte injury by the following mechanisms. First, Tau phosphorylation in podocytes may lead to microtubule depolymerization, resulting in cytoskeleton destruction. The transport of cell organelles and large molecules, both of which rely on cytoskeleton, might be blocked [24]. Primary and secondary podocyte extensions are maintained by microtubules whose depolymerization may be related with observed simplification. Moreover, neuroscience research have shown that the accumulation of p-Tau in neurons may cause neuron dysfunction and neuropathology [39]. Similarly, the toxic p-Tau increasing in podocytes resembles the effect in neurons. Thus, the abnormally upregulated p-Tau protein might induce podocyte dysfunction. As LiCl treatment prevented podocyte pathology, luteolin application conferred a protective effect similar to that of LiCl in the chronic METH injury model (Figures 6 and 7).

To understand the mechanism of Lut in attenuating METH-induced nephropathy, we used an in vitro model. We found that protection of METH nephropathy by Lut may be through the GSK3*β*-Tau phosphorylation pathway (Figure 8).

**Figure 9:**
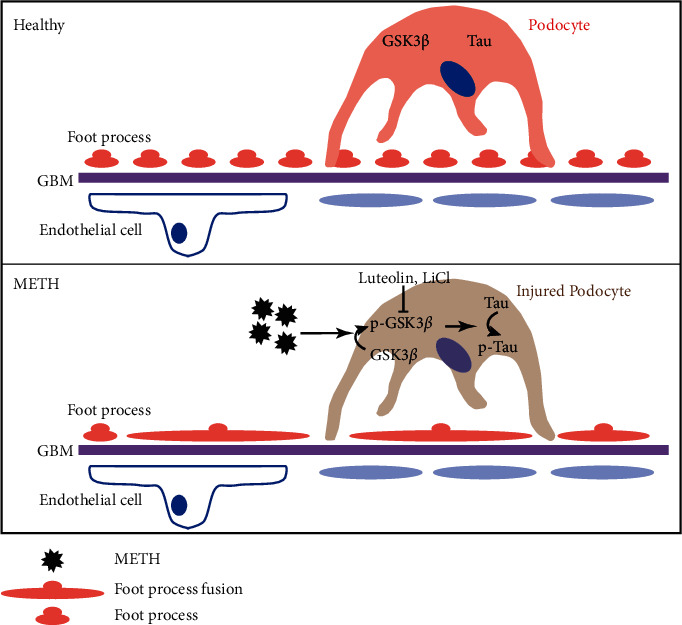
Schematic illustration of the mechanism for p-Tau to mediate METH-induced podocyte pathology. Upon METH treatment, the activated GSK3*β* (phosphorylated GSK3*β*) could lead to Tau phosphorylation. The phosphorylated Tau may trigger podocyte pathology including podocyte simplification, foot process effacement, and podocyte specific protein loss. Inhibiting GSK3*β* activation by LiCl could alleviate the podocyte injury induced by METH. The luteolin could prevent the podocyte pathology, and it might be p-Tau dependent.

Taking together, the results of this study demonstrate that p-Tau mediates METH nephrotoxicity, and inhibiting Tau phosphorylation could prevent nephropathy. Luteolin could protect METH nephrotoxicity including macrophage infiltration, glomerulus fibrosis, podocyte simplification, foot process effacement, and podocyte morphology abnormalities in the chronic METH mice model. The renal-protective effect seems to be dependent on Tau phosphorylation inhibition (Figure 9).

## 5. Conclusion

We have found that chronic METH might induce glomerular fibrosis, podocyte pathology through Tau phosphorylation. Luteolin could prevent the podocyte pathology through inhibiting p-Tau level increase. Collectively, luteolin treatment prevented nephropathy, suggesting that luteolin might be a promising candidate of a preventive role in METH-induced kidney diseases. Future studies have to test the effect of luteolin on other types of nephropathies.

## Figures and Tables

**Table 1:** Dosing schedule of methamphetamine (METH) treatment (mg/kg).

Day	1	2	3	4	5	6	7	8	9	10	11	12	13	14
8:00	1.0	1.0	1.0	1.0	1.5	1.5	2.0	2.0	2.5	3.0	3.5	4.0	4.5	5.0
10:00				1.0	1.5	1.5	2.0	2.0	2.5	3.0	3.5	4.0	4.5	5.0
12:00				1.0	1.5	1.5	2.0	2.0	2.5	3.0	3.5	4.0	4.5	5.0
14:00		1.0	1.0	1.0	1.5	1.5	2.0	2.0	2.5	3.0	3.5	4.0	4.5	5.0

**Table 2:** Primary antibodies used in the study.

Antibody	Host	Distributor	Working dilution	
			IHC	WB
p-Tau (ser396)	Rabbit	Abcam, ab109390	1:300	1:1000
p-GSK3*β* Y216	Rabbit	Abcam, ab75745	1:500	1:2000
Podocalyxin-like protein 1	Mice	Santa Cruz, sc23903	1:200	1:1000
Synaptopodin	Rabbit	Abcam, 224491	1:200	1:1000
Iba-1	Rabbit	Abcam, 178846	1:200	—

## Data Availability

All processed data and models used during the study are available from the corresponding authors by request. But the raw data required to reproduce these findings cannot be shared at this time as the data also form part of an ongoing study.
